# Parenting Stress Following a Neonatal Intensive Care Unit Hospitalization: A Longitudinal Study of Mothers and Fathers

**DOI:** 10.3390/ijerph21080970

**Published:** 2024-07-25

**Authors:** Corinna C. Klein, Nicole M. McDonald

**Affiliations:** Semel Institute of Neuroscience and Human Behavior, David Geffen School of Medicine, University of California, Los Angeles, CA 90095, USA; nmcdonald@mednet.ucla.edu

**Keywords:** NICU, parent stress, preterm birth, perinatal stressors

## Abstract

A neonatal intensive care unit (NICU) hospitalization can add significant stress to the postpartum period. Parents experience isolation and uncertainty, which can affect their capacity to bond with their new baby. Understanding how stress is shaped by and changes following a NICU experience will help in developing supports for these families. We examined patterns of parenting stress over the first year of life following a NICU stay to better understand changes in stress, differences in maternal and paternal stress, and how medical and developmental variables impact parent stress. Parents of infants (*n* = 51) who had experienced a NICU hospitalization and met criteria for California’s High-Risk Infant Follow-Up program completed assessments at 6, 9, and 12 months. A comparison group (*n* = 38) from a historic dataset included parents of infants born full term without medical complications. NICU parents reported higher levels of parenting stress at 6 months, but not 12 months, with mothers and fathers reporting similar stress levels. Parenting-related stress was found to be relatively stable and consistent over this period. Among NICU parents, lower developmental level at 12 months was associated with more distress in interacting with their child. These findings highlight the importance of monitoring parenting stress following discharge from the NICU and developing interventions for supporting parents of NICU graduates showing developmental delays.

## 1. Introduction

Parental mental health is a fundamental component of neonatal care, with particular importance for families who have faced perinatal stressors such as a neonatal intensive care unit (NICU) stay. The NICU experience can complicate the transition to parenthood, increasing feelings of isolation and distress [1]. Parents who have experienced a NICU stay have been found to exhibit high levels of mental health symptoms, including depression, anxiety, and post-traumatic stress [2,3]. Parents whose babies experience NICU stays are under high levels of stress at the onset of parenthood [4], and their emotional responses to a NICU stay or preterm birth can impact their parenting and bond with their baby [5,6]. Parental mental health can have a significant impact on an infant’s psychological, social, and cognitive development [7], and parenting stress can persist for several years following the birth of a high-risk infant [8].

Researchers and neonatologists have increasingly called for early screening of psychological symptoms in new parents going through a NICU stay [9]. Multiple studies have attempted to identify parent and infant factors that are predictive of caregiver stress following a NICU stay. Ionio and colleagues [9] found that for both mothers and fathers, younger age predicted higher stress in NICU parents, with paternal stress predicted by lower gestational age and maternal stress by their baby’s need for respiratory support. In a study of 162 parents of infants in the NICU, gestational age, length of stay, extreme prematurity, and a cardiovascular diagnosis were found to predict parent stress during their baby’s NICU stay [10]. Turner and colleagues [11] found in a sample of 73 Australian parents that older age, very premature birth, and twin birth were associated with higher parent stress in the NICU. Attempts to identify predictors of parent stress have had varying results, without pointing to consistent predictors of caregiver stress. Additionally, most of these studies focus on caregiver stress during or immediately following a NICU stay, without assessing how this stress may evolve over time. Studies assessing changes in stress have found different stress responses in mothers and fathers. One study comparing maternal and paternal stress in parents of preterm infants in Sweden found that maternal stress decreased from 8 weeks to 6 months, while paternal stress increased from 6 to 12 months [12]. The study found that gestational age (<32 weeks), twins, and parental health also impacted stress level [12].

While it is evident that the NICU can be a stressful experience, there is limited clarity regarding what predicts subsequent parenting stress and how patterns of stress evolve over time. Research has focused on variables related to the NICU stay or birth, but has not assessed whether infant development following the NICU may impact caregiver stress. We followed families of NICU graduates over the first year of life. A non-NICU comparison group included families recruited as part of a historic community sample. We first aimed to investigate patterns of parenting stress in the first year of life in parents of infants with and without a NICU stay. We then assessed how levels of parenting stress change over the first year of life in both groups. As a third aim, we investigated whether variability in parenting stress could be explained by infant developmental or medical factors. Finally, we evaluated differences in parenting stress reported by mothers and fathers for a subset of families with data from both parents. The overall objective of the current study was to investigate parenting stress among NICU families at three timepoints following a NICU stay: 6, 9, and 12 months.

## 2. Methods

### 2.1. Participants and Procedures

The current study was part of a larger longitudinal study that followed infants after a NICU stay, the Neurodevelopment and Early Social–Emotional Trajectories in NICU Graduates (NESTING) study. Participants were recruited directly from the High-Risk Infant Follow-up (HRIF) Clinic at the University of California, Los Angeles, where eligible families were approached by a research associate and provided written and verbal information about the study. Infants (*n* = 51) had experienced a NICU hospitalization following birth and met criteria for the California HRIF program as defined by California Children’s Services. High-risk criteria include gestational age under 32 weeks, birthweight at or under 1500 g, neurological or cardiac issues, and other medical concerns and interventions that indicate increased medical risk. Infants with vision or hearing impairments or who were not medically stable at the time of visit were excluded from the NESTING study. Families who expressed interest in participating were contacted for screening by a research team member, and those who qualified were scheduled for an initial visit. In families where both twins qualified for the study (*n* = 2), data from only one twin (selected randomly) was included to maintain independence. Sample demographics can be found in Table 1. 

Data were collected from participating families at 6 and 9 months via a HIPAA-compliant Zoom platform and at 12 months via a hybrid (remote/in-person) format. Data for the NESTING study were collected between 2021 and 2023. Visits were scheduled using chronological age for term children and adjusted age via expected due date for preterm children (those born < 37 weeks). Parents completed online measures and participated in clinical interviews with trained clinicians. Additional information was gathered from infants’ medical charts. The study was approved by the UCLA Medical Institutional Review Board 3 (MIRB3), and all parents provided informed consent, including a separate consent to access medical records.

Data for the non-NICU comparison group (*n* = 38) were gathered from a historic dataset (2015–2016) recruited through the Yale-New Haven Hospital Newborn Nursery, where families received a brochure and had the option to provide contact information for study staff. Additional infants were recruited through word of mouth. Infants born premature, with low birth weight, or with major medical, vision, or hearing problems were excluded. Demographics for the comparison group can be found in Table 1. Data were collected at 3, 6, and 12 months via in-person assessment, with data from the latter two visits used in this study. The study was initially approved by the Yale Human Investigations Committee and has current approval through UCLA MIRB3. 

### 2.2. Measures

#### 2.2.1. Medical and Demographic Data 

A trained research associate conducted parent interviews at each child’s 6-month visit, which included gathering infant and parent demographic data. Medical history was also gathered during these interviews and subsequently confirmed via medical record review. Length of NICU stay, gestational age, and HRIF criteria were gathered for each child in the NICU group.

#### 2.2.2. Parenting Stress 

The Parenting Stress Index, Fourth Edition Short Form (PSI-4-SF) was used to evaluate parenting-related stress. The PSI-4-SF [13] is a parent report measure for parents of children aged 1 month to 12 years that includes 36 items that assess three domains of parenting stress: Parental Distress, Parent-Child Dysfunctional Interaction, and Difficult Child, which contribute to a Total Stress score. Higher scores indicate more parenting stress. The PSI-4-SF also yields a Defensive Responding score, which indicates the extent to which a caregiver may minimize parenting challenges to convey a positive impression. A score of 10 or less indicates defensive responding. Scores above the 85th percentile are typically considered clinically significant. 

#### 2.2.3. Developmental Abilities 

The Mullen Scales of Early Learning (MSEL) is a standardized assessment that assesses the development abilities of children from birth to 68 months. The measure yields five domain scores (gross motor, fine motor, visual reception, receptive language, and expressive language), as well as a composite cognitive ability score (the Early Learning Composite [ELC]), which includes all domain scores except gross motor. MSEL data were available for 80 infants at 12 months (*n* = 42 for NICU, *n* = 38 for non-NICU). Scores were calculated using the expected due date for preterm children.

### 2.3. Data Analysis Plan

All analyses were conducted using SPSS 29. To assess patterns of parenting stress in the year after a NICU stay, means and standard deviations for all scales at each timepoint were calculated. To assess change over time, a repeated measures ANOVA was run with NICU stay as the between-subjects factor and time as the within-subjects variable. The same model was then run with parental education as a covariate. Due to the small sample size, parental education was included as a binary variable, with high school and some college collapsed, and college degree and graduate education collapsed. For our third aim, to assess whether variability in parenting stress could be explained by developmental factors, bivariate Pearson’s correlations were calculated between parenting stress and the MSEL at 12 months across both groups. To investigate whether parenting stress varied by medical factors (length of NICU stay, gestational age), additional correlations were run at 6 months. Finally, we assessed the degree to which parenting stress in mothers and fathers correlated with each other and compared levels of parenting stress across mothers and fathers by running a paired-sample t-test in a subset of the sample for whom we had data for both parents (*n* = 39). Aside from this final analysis, analyses were run using data for the parent who identified as the primary caregiver (NICU group: 47 moms, 3 dads; non-NICU group: 35 moms, 2 dads).

## 3. Results

### 3.1. Participant Demographics

Differences between demographic variables were investigated using t-tests for continuous variables, chi-square for categorical variables with adequate cell size, and Fisher’s exact test for variables with small cells (*n* < 5). As seen in Table 1, the groups had similar developmental profiles (Mullen ELC) and were balanced in terms of sex. Significant differences existed between groups in terms of infant ethnicity and parent education. Differences in infant race and parent marital status approached significance.

### 3.2. Aim 1: Patterns of Stress Among NICU and Non-NICU Parents in the First Year of Life 

Descriptive statistics were examined to understand patterns of parenting stress over the first year of life. Skewness and kurtosis results indicated roughly normal and non-skewed distributions for the full sample, as well as for the NICU and non-NICU groups individually. The PSI Total Stress score and all subscale items were internally consistent across ages and groups (α 0.674–0.952). Parents in both groups displayed relatively high rates of defensive responding (see Table 2). Parents reported Total Stress scores below the clinical cutoff at all timepoints. 

### 3.3. Aim 2: Change in Parenting Stress from 6 to 12 Months

Intercorrelations across time were run for Total Stress and PSI-4-SF subscales at 6 and 12 months across both groups. Total Stress was strongly correlated, r(69) = 0.661, *p* < 0.001, 95% CI [0.506, 0.775], from 6 to 12 months, suggesting consistency over time. Follow-up analyses of subscales indicated moderate-to-strong correlations within each subscale (rs = 0.551–0.677). Follow-up analyses within each group suggested that the consistency in parenting stress scores was primarily driven by findings within the NICU group (rs = 0.546–0.728). Scores were slightly less consistent over time within the non-NICU group (rs = 0.308–0.556). Within the NICU group, Parental Distress was most strongly correlated, while in the non-NICU group, it was the most weakly correlated.

**Figure 1:**
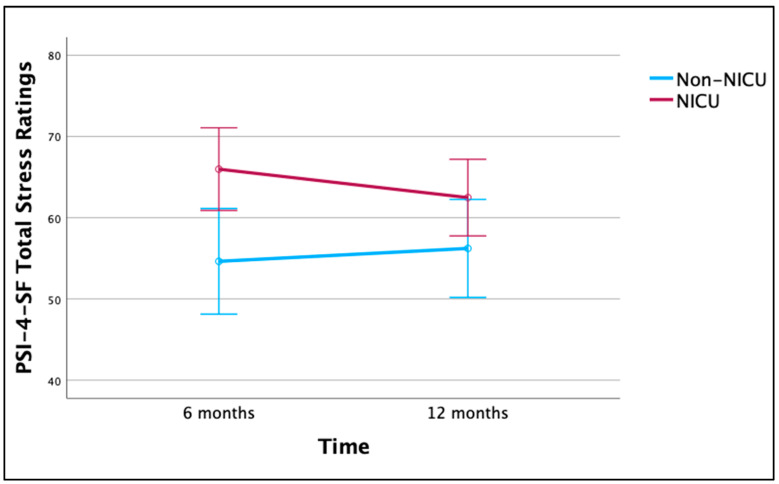
Total Stress (raw score) for NICU and non-NICU families over time. Note: figure presents estimated marginal means from ANOVA with 95% confidence intervals.

A repeated-measures ANOVA was conducted to evaluate the effect of group (NICU vs. non-NICU) and time (6 vs. 12 months) on Total Stress for families with data at both timepoints (*n* = 71). The main effect of time on parenting stress was not significant F(1,69) = 0.320, *p* = 0.573, η_p_^2^ = 0.005, suggesting consistent levels from 6 to 12 months. The main effect of group on parenting stress was significant, F(1,69) = 5.920, *p* = 0.018, η_p_^2^ = 0.079. There was no significant interaction between time and group, F(1,69) = 2.281, *p* = 0.136, η_p_^2^ = 0.032 (see Figure 1). Findings were similar when PSI-4-SF subscale scores were analyzed. 

The same model was run with parental education (high school and/or some college vs. college degree and above) as a covariate. The main effect of time on parenting stress remained not significant, F(1,68) = 0.827, *p* = 0.366, η_p_^2^ = 0.012. The main effect of group on parenting stress remained significant, F(1,68) = 7.222, *p* = 0.009, η_p_^2^ = 0.096. The main effect of parent education was not significant, F(1,68) = 1.627, *p* = 0.206, η_p_^2^ = 0.023. There were no significant interactions between time and group, F(1,68) = 3.425, *p* = 0.069, η_p_^2^ = 0.048, or between time and parent education, F(1,68) = 2.218, *p* = 0.141, η_p_^2^ = 0.032. However, in both cases, there was a small-to-moderate effect.

### 3.4. Aim 3: Explaining Variability in Parenting Stress Using Developmental and Medical Factors

**Figure 2:**
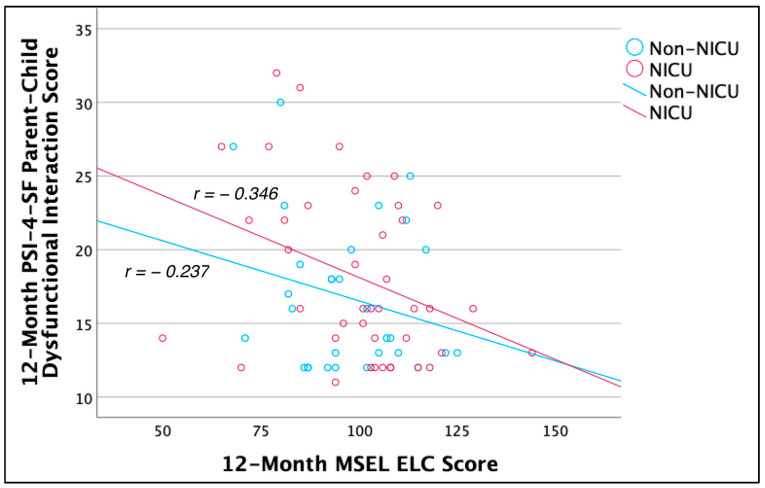
12-month Parent–Child Dysfunctional Interaction (raw score) and Mullen Early Learning Composite.

Correlations for the whole sample (NICU and non-NICU parents) were examined between 12-month PSI-4-SF Total Stress and parenting stress subscales and 12-month child developmental level (Mullen—ELC). A significant correlation was found between ELC and Parent–Child Dysfunctional Interaction scores, r(70) = −0.296, *p* = 0.012, 95% CI [−0.494, −0.069], with children of parents reporting more dysfunctional interactions showing lower developmental abilities. No significant correlations were found between ELC and the other scales (rs = −0.077–−0.176). To better understand the correlation between ELC and Parent–Child Dysfunctional Interaction across the full sample, post hoc correlations were conducted within each group. Within NICU parents, ELC and Parent–Child Dysfunctional Interaction were moderately negatively correlated, r(40) = −0.346, *p* = 0.025, 95% CI [−0.588, −0.047]. Within non-NICU parents, the correlation was in the same direction but slightly weaker and did not reach significance, r(28) = −0.237, *p* = 0.208, 95% CI [−0.550, 0.135], suggesting that this effect was primarily driven by the NICU parents (see Figure 2).

The relationship between medical factors (days in NICU and gestational age) and parenting stress was examined at 6 months for the NICU group. Neither length of stay in the NICU (rs = 0.122–0.198) nor gestational age (rs = −0.001–0.066) was correlated with any domains of parenting stress at 6 months.

### 3.5. Aim 4: Investigate Differences in Maternal and Paternal Parenting Stress

Descriptive statistics for maternal and paternal parenting stress for a subgroup (*n* = 39) of parents who provided both maternal and paternal parenting stress scores at 6 months are present in Table 3. For 10 families who did not complete the PSI-4-SF at 6 months, scores from 9 months were used. Mothers and fathers reported similar levels of Total Stress, t(38) = −0.618, *p* = 0.540, d = −0.099. Total Stress and all subscales were moderately correlated between parents (see Table 4). 

## 4. Discussion

The current study investigated patterns of parenting stress over the first year of life following a NICU stay. Parents of NICU graduates reported more parenting-related stress than parents whose infants did not experience NICU stays. Parenting stress scores were relatively stable and consistent from 6 to 12 months, with parental distress particularly consistent in the parents of NICU graduates. Although the interaction between time and group (NICU vs. non-NICU) was not significant, the effect size was small to medium, suggesting that with a larger sample, different patterns of stress over time may be found between NICU and non-NICU families. A similar pattern was observed when parental education was included in the model, with small-to-medium effect sizes observed in the interactions between both time and group and time and education. Among NICU parents, lower developmental level in their 12-month-old was associated with more distress related to interactions with their child, while past medical characteristics (gestational age and length of NICU stay) were unrelated to parenting stress. Mothers and fathers reported similar stress levels in the first year of their child’s life. These findings have implications for supporting parents following a NICU stay, and for identifying parents in need of intervention.

Parents of infants in this study who had extended NICU stays reported higher parenting-related stress when compared to a non-NICU historic sample. This finding was mainly driven by differences in parenting stress at 6 months of age, rather than 12 months. These findings are somewhat in contrast with previous studies, which observed similar levels of stress (measured by the PSI-SF) between mothers of term and preterm infants (born at 24–30 weeks) at 4 months yet found larger stress disparities at 1 year (corrected for prematurity) [14,15]. The difference in these findings could be due to the current study’s inclusion of a wider variety of medical conditions leading to a NICU stay. The use of a historical sample may also have contributed to observed differences in stress. Cohort effects, including the study’s timing and demographic differences between the two groups, may have impacted our findings. While the presence or absence of a NICU stay was the primary difference of interest between our groups, the groups were also different demographically, with non-NICU parents reporting higher levels of education and less ethnic and racial diversity.

Parents in this study reported stress under the recommended 85th percentile clinical cutoff. However, it has been suggested that a lower cutoff score may be more accurate when working with high-risk families and infants. A study evaluating the PSI-4-SF with high-risk mothers and their 12- to 15-month-old infants recommended a lower cutoff score (73rd–77th percentile) to accurately identify mothers with depressive symptoms or infants with emotional and behavioral challenges [16]. Barroso and colleagues [16] suggested that a higher clinical cutoff may exclude parents in need of services. A study assessing maternal stress in a community sample of 6- to 18-month-old infants, in which medically complex or developmentally delayed infants were excluded, found a mean at the 29th percentile for maternal stress at 6 months and the 32nd percentile at 18 months [17]. Subclinical scores on the PSI-4-SF may be common in infants both with and without medical diagnoses or early-life stressors, such as a NICU stay.

High levels of defensive responding in the current sample may also have contributed to all parents scoring below the clinical threshold. This response pattern has similarly been found in parents of 4-month-old infants with congenital heart disease [18]. The authors hypothesized that social desirability and reluctance to acknowledge their child’s medical condition may contribute to defensiveness. It is possible that parents who have experienced neonatal or early-life medical stressors tend to minimize parenting-related stress in the first year of life. However, in the current study, non-NICU parents also demonstrated high rates of defensive responding. Since data for the current study were collected as part of broader developmental infant assessments, it is also possible that parents felt compelled to minimize issues, wanting their infants to be positively evaluated. It is also possible that measure items are better suited to older-aged children. Together, there is evidence that the PSI may underestimate parenting-related stress in parents of infants, suggesting that scores may be meaningful when making comparisons, but less helpful for determining individual need.

Parenting stress was found to be generally stable and consistent over time among both groups. Within the NICU group, the strongest correlation was seen in parental distress, or the parent’s perception of their own competence, capability, and level of support in their parenting role. While NICU-related parenting stress may naturally decrease over time, and perceptions of one’s child (as seen in the difficult child and parent–child interaction subscales) shift along with a child’s growth and development, parental self-perceptions may remain more consistent. This suggests that parent distress at 6 months may be a helpful barometer for subsequent distress within NICU parents when assessing whether support or intervention is needed. Parental distress was additionally the only subscale in which some NICU parents scored above the clinical cutoff. Parent perceptions of their child as difficult to care for and their satisfaction with their interactions with their child may vary more in the first year of life, as infants are developing and changing at high rates. It is also possible that increases in child-related stress may emerge as children reach toddlerhood and challenging behaviors increase, versus the more basic challenges of infancy (e.g., feeding and, sleeping).

Developmental level based on in-person standardized testing at one year was correlated only with parenting stress related to their interactions with their child, suggesting a potential impact of developmental delays on the perceived quality of parent–child interactions (although directionality is unclear). This correlation appeared slightly stronger for the NICU sample, suggesting that parents who have experienced a NICU stay may be more alert to their child’s development and distressed by any perceived delays. However, given that there is an overlap in the confidence intervals for this correlation, interpretations should be made with caution, and would require a larger sample to confirm. Other birth or NICU characteristics (gestational age, time in NICU) were not correlated with later parenting stress. While prior studies have found that each of these variables impact parent stress during the NICU stay [9,10], the current study indicates that the impact of these variables may decrease with time. 

Mothers and fathers in our study reported similar levels of distress. Parenting-related stress was moderately correlated, with the strongest correlation between their perceptions of their child as difficult. This indicates some agreement between parents on how difficult they find their child to be and how stressful it is to parent that child. Prior research has suggested that mothers of preterm infants experience greater stress than fathers while experiencing a NICU stay [19] and has identified different predictors of stress in mothers and fathers following a NICU stay [19]. Although it is possible that parent stress differs during a NICU stay, particularly given its temporal proximity to the birth experience, maternal and paternal stress may become more similar over time. Initial screeners during the NICU stay may not adequately reflect how stress will evolve following discharge.

Consistency between maternal and paternal stress reaffirms the importance of family-focused interventions to address stress following a NICU stay. Many types of interventions have been developed and tested to support families transitioning home after a NICU stay, including home visits, telehealth or video support, app-based support, and providing sleep and feeding support in various formats [20]. Recommendations during and after discharge include both psychological support for parents and concrete education on child development, sleep, feeding, and medical concerns [21]. Additional interventions focused on increasing parental confidence, sensitivity, attunement, and sense of competence as a parent include the Newborn Behavioral Observation, which is a promising option for infant–parent dyads following a NICU stay [22,23]. Although this intervention has not been delivered to infants following a NICU stay, it has been found to increase learning related to infancy and a baby’s needs in the first few months of life, and to increase maternal sensitivity [22,24]. Given developmental concerns following a NICU stay, interventions that target parental concerns and uncertainty about child development, while promoting parent–child bonding, sensitivity, and attunement, may be particularly helpful for this population. 

### 4.1. Limitations and Future Directions

This study uniquely examined the progression of parenting-related stress in a cohort of parents whose infants experienced a NICU stay over the first year of life. We utilized a historical comparison group as a point of contrast for interpreting findings from the NICU parents. While this comparison provided unique insights, results related to group differences should be interpreted with caution. Our non-NICU comparison group differed across several demographic variables, and data collection for that group occurred prior to the COVID-19 pandemic. Conversely, our NICU sample was collected during the pandemic. It is possible that the group differences observed could also have been influenced by broader stressors experienced related to the timing of data collection or composition of the group. Our analysis was also limited by a relatively small sample size, particularly with regard to mother/father comparisons, so non-findings, in particular, should be interpreted with some caution. Future studies would benefit from concurrent comparisons across matched groups to confirm the current results. We will continue to follow these families through age two, allowing for the future evaluation of parenting stress as the infants reach toddlerhood and behavioral challenges increase and developmental delays become more evident. This longitudinal evaluation will provide insight into parenting stress during different developmental periods and highlight whether stress peaks at a particular age.

### 4.2. Conclusions

This study provides additional insight into patterns of parenting stress in the first year of life following a NICU stay. While parents may underreport their stress, partly due to relief that their child is home and no longer in a precarious condition, they do show variations in stress and potentially higher stress than families who have not experienced a NICU stay. Additionally, their stress levels may be uniquely impacted by their child’s early development, with heightened attentiveness to developmental level following their infant’s hospitalization. It may be that measuring general stress levels early on would elicit more insight into subsequent parenting stress, rather than measuring infant-focused stress, which shows more variability with time. When assessing parent stress and the need for intervention, screenings should be conducted over the first year and beyond, rather than solely during a NICU stay, and should assess both mothers and fathers. Parents who have experienced a NICU stay may also benefit from increased education from providers regarding developmental expectations to mitigate worries that may impact their ability to enjoy interactions with their child, while enabling them to accurately detect areas of concern.

## Figures and Tables

**Table 1:** Participant Characteristics.

	NICU *n* = 51	Non-NICU *n* = 38	*p*-Value
Gestational Age in weeks	33.19 (5.26)		
Infant birth weight (g)	1892.06 (971.126)		
Days in NICU	57.31(44.69)		
Number of older siblings	1.08 (1.32)	0.89 (1.07)	0.214
12-Month Developmental Quotient (MSEL ELC)	99.74 (17.98)	98.37 (14.74)	0.712
Infant sex			0.335
Female	24 (47.1%)	14 (36.8%)	
Male	27 (52.9%)	24 (63.2%)	
Infant race			0.060
White	29 (56.9%)	27 (71.1%)	
Black/African American	10 (19.6%)	2 (5.3%)	
Multiracial	4 (7.8%)	7 (18.4%)	
Asian/Pacific Islander	7 (13.7%)	2 (5.3%)	
Infant ethnicity (Hispanic/Latinx)	25 (49.0%)	2 (5.3%)	<0.001
Maternal Education			0.014
High School or less	8(15.7%)	5(13.2%)	
Some college	15 (29.41%)	5 (13.2%)	
College degree	14 (27.45%)	6 (15.8%)	
Graduate/professional degree	12 (23.5%)	22 (57.9%)	
Paternal Education			0.013
High School or less	17 (33.3%)	3 (7.9%)	
Some college	8 (15.7%)	5 (13.2%)	
College degree	11 (21.6%)	11 (28.9%)	
Graduate/professional degree	11 (21.6%)	17 (44.7%)	
Marital Status			0.142
Single Parent	7 (13.7%)	2 (5.3%)	
Other/Separated	3 (5.9%)	1 (2.6%)	
Married	34 (66.7%)	32 (84.2%)	
Living Together	7 (13.7%)	3 (7.9%)	
HRIF Primary Qualifying Condition			
Preterm (<32 weeks)	22 (43.14%)		
Low Birthweight (≤1500 g, ≥32 weeks)	8 (15.7%)		
Major Neuro Injury (≥1500 g, ≥32 weeks)	10 (19.6%)		
Cardiac (No Neuro Injury, >1500 g, ≥32 weeks)	6 (11.76%)		
Other (No Neuro/Cardiac, >1500 g, ≥32 weeks)	5 (9.8%)		

Note: Developmental scores based on the Early Learning Composite (ELC) measured via the Mullen Scales of Early Learning (MSEL). Nine NICU participants were missing data on 12-month developmental level. Race was missing for one Hispanic participant. Number of siblings was missing for 2 participants. To maintain sufficient cell size for group comparisons, some demographic groups were collapsed. For paternal education, high school and some college were combined. Within marital status, married was combined with living together and single parent with other/separated. HRIF Primary Qualifying Condition presented in priority order.

**Table 2:** Means and SD for PSI-4 subscales.

Scale	Mean (SD) or % Above Cutoff				
	NICU			Non-NICU	
	6 Months *n* = 50	9 Months *n* = 48	12 Months *n* = 45	6 Months *n* = 35	12 Months *n* = 30
Defensive Responding	26%	35.4%	37.8%	46%	43%
Parental Distress	25.14 (8.68)	22.79 (8.17)	23.38 (8.35)	20.54 (4.80)	20.87 (5.41)
Parent–Child Dysfunctional Interactions	19.30 (6.05)	18.50 (5.32)	18.29 (5.83)	16.02 (4.60)	16.77 (5.04)
Difficult Child	21.14 (6.55)	20.94 (5.89)	20.31 (5.44)	18.20 (5.03)	19.23 (4.20)
Total Stress	65.58 (19.04)	62.23 (17.12)	61.98 (17.75)	54.77 (12.50)	56.87 (12.67)

Note: All scale scores are raw scores.

**Table 3:** Maternal and paternal means and SD for PSI-4-SF subscales at 6–9 months.

	NICU *n* = 23		Non-NICU *n* = 16	
	Mothers	Fathers	Mothers	Fathers
Parental Distress	24.09 (8.04)	23.04 (9.07)	20.00 (6.76)	20.81 (6.42)
Parent–Child Dysfunctional Interactions	18.00 (5.31)	17.96 (4.97)	14.44 (3.18)	16.00 (3.39)
Difficult Child	21.74 (6.46)	22.70 (7.80)	16.81 (4.58)	18.94 (5.12)
Total Stress	63.83 (17.26)	63.69 (19.83)	51.25 (12.54)	55.75 (12.53)

**Table 4:** Correlations between maternal and paternal PSI-4-SF scores.

	Parent Distress	Parent–Child Dysfunctional Interaction	Difficult Child	Total Stress
Pearson’s Correlation	0.424 **	0.396 *	0.442 **	0.450 **
95% CI	0.125–0.652	0.092–0.632	0.147–0.665	0.156–0.670

** Correlation is significant at the 0.01 level. * Correlation is significant at the 0.05 level.

## Data Availability

The data presented in this study are available on request from the corresponding author (cckleins@mednet.ucla.edu).
